# Characterization and Quantification of the Compounds of the Ethanolic Extract from *Caesalpinia ferrea* Stem Bark and Evaluation of Their Mutagenic Activity

**DOI:** 10.3390/molecules191016039

**Published:** 2014-10-08

**Authors:** Carlos César Wyrepkowski, Daryne Lu Maldonado Gomes da Costa, Adilson Paulo Sinhorin, Wagner Vilegas, Rone Aparecido De Grandis, Flavia Aparecida Resende, Eliana Aparecida Varanda, Lourdes Campaner dos Santos

**Affiliations:** 1 Organic Chemistry Department, Institute of Chemistry, São Paulo State University (UNESP), Araraquara 14800-900, Brazil; 2 Mato Grosso Federal Institute (IFMT), Cuiabá 78043-400, Brazil; 3 Institute of Natural, Human, and Social Sciences, Mato Grosso Federal University (UFMT), Sinop 78557-267, Brazil; 4 Experimental Campus of São Vicente, São Paulo State University (UNESP), São Vicente 11350-000, Brazil; 5 Department of Biological Sciences, Faculty of Pharmaceutical Sciences, São Paulo State University (UNESP), Araraquara 14801-902, Brazil

**Keywords:** *Caesalpinia ferrea*, HPLC-DAD quantification, HPLC/ESI-IT-MS, hydrolyzable tannins, mutagenic activity

## Abstract

*Caesalpinia ferrea* Martius has traditionally been used in Brazil for many medicinal purposes, such as the treatment of bronchitis, diabetes and wounds. Despite its use as a medicinal plant, there is still no data regarding the genotoxic effect of the stem bark. This present work aims to assess the qualitative and quantitative profiles of the ethanolic extract from the stem bark of *C*. *ferrea* and to evaluate its mutagenic activity, using a *Salmonella*/microsome assay for this species. As a result, a total of twenty compounds were identified by Flow Injection Analysis Electrospray Ionization Ion Trap Mass Spectrometry (FIA-ESI-IT-MS/MS^n^) in the ethanolic extract from the stem bark of *C*. *ferrea*. Hydrolyzable tannins predominated, principally gallic acid derivatives. The HPLC-DAD method was developed for rapid quantification of six gallic acid compounds and ellagic acid derivatives. *C*. *ferrea* is widely used in Brazil, and the absence of any mutagenic effect in the *Salmonella*/microsome assay is important for pharmacological purposes and the safe use of this plant.

## 1. Introduction

*Caesalpinia ferrea* Mart. is a leguminous plant found in the north and northeastern semi-arid region of Brazil. It is more commonly known in Brazil as *pau-ferro* or *juca* and is widely used in folk medicine [1,2]. *C*. *ferrea* underwent a reclassification in 2009 and is now known as *Libidibia ferrea* [3]. However, due to chemical and pharmacological data for *C*. *ferrea* being reported in the literature, it was not necessary to change the name for this work.

Infusions of *C*. *ferrea* have been used for years to treat various disorders, including in analgesic and anti-inflammatory treatments [4,5], antiulcerogenic treatments [6], cancer chemopreventives [7], antimicrobial applications [8] and healing treatments [9]. In Brazil, the tea from the stem bark of *C*. *ferrea* has been widely used in folk medicine to treat diabetes mellitus [10]. 

The hypoglycemic properties of the aqueous extract of the stem bark of *C*. *ferrea* were investigated by Vasconcelos *et al*. [11], and the mechanisms by which the extract reduces the blood glucose levels were elucidated.

A preliminary phytochemical study of the stem bark of *C*. *ferrea* revealed the presence of flavonoids, saponins, tannins, coumarins, steroids and phenolic compounds [11,12]. Additionally, gallic acid, catechin, epicatechin and ellagic acid have also been identified by HPLC [11]. Pauferrol A, a unique chalcone derivative, was isolated from the stems, and the structure was determined to be a chalcone trimer fused by a cyclobutane ring [13]. Chalcone dimers, pauferrol B and pauferrol C were also isolated [14]. 

A review of the healing power of plants and a return to natural remedies is an absolute requirement of our time, because these herbal medicines have natural compounds that can promote health [15].

Many plants have been described in the literature as having mutagens in their constitution [16,17,18].

Interest in *C*. *ferrea* is justifiable, because of its potential medicinal value. Apart from the health benefits of herbal medicines, little is known about their potential mutagenic properties. Mutagenicity may lead to severe genetic alterations and cancer at doses much lower than what is necessary to display acute toxicity. Despite the popular use of *C*. *ferrea* as a medicinal plant, there are still no data regarding its genotoxic effects.

Although *C*. *ferrea* is widely used in folk medicine, to our knowledge, there is no information available for a comprehensive identification of its chemical composition or quantification by a validation method or study of the mutagenic activity of the ethanolic extract from the stem bark of this species. Therefore, the aim of the present study was identified, or tentatively identified, as being the determination of the constituents and quantification of certain compounds in the ethanolic extract from the stem bark, as well as evaluation of the mutagenic activity using a *Salmonella*/microsome assay.

## 2. Results and Discussion

### 2.1. Identification of Constituents by FIA-ESI-IT-MS/MS^n^

**Figure 1:**
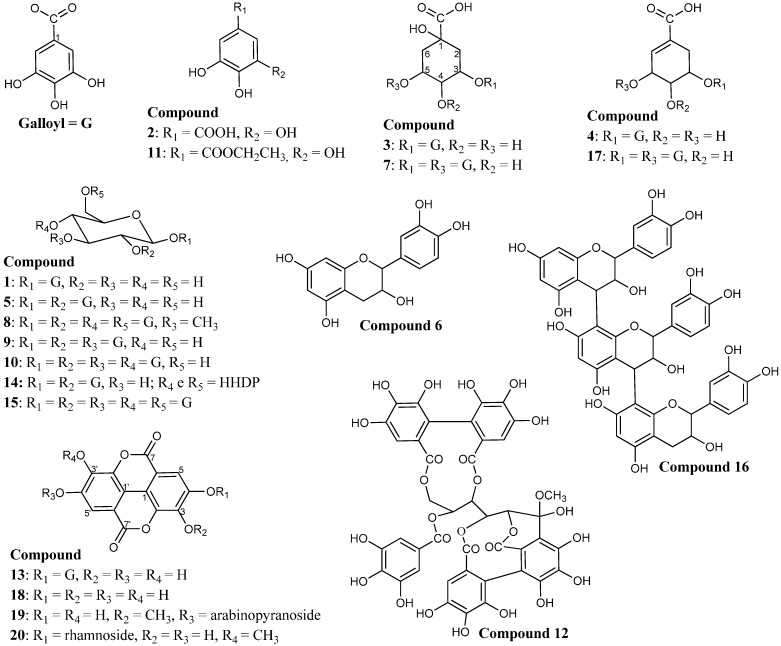
Structures of the twenty constituents identified in the ethanolic extract of the stem bark of *C*. *ferrea*.

In total, twenty-six compounds were identified by FIA-ESI-IT-MS/MS^n^ (Figure 1) in the ethanolic extract of the stem bark of *C*. *ferrea*. Data concerning the identification of the peaks are shown in Table 1, in which we report the retention time, UV-Vis absorptions and electrospray ionization mass spectrometry in negative ion mode for all of the compounds detected. Since this is the first experiment for the chemical characterization of most of the constituents in the ethanolic extract from the stem bark of *C*. *ferrea*, it was necessary to characterize them completely. 

**Table 1:** HPLC/ESI-IT-MS data of the compounds detected in the ethanol extract from the stem bark of *C*. *ferrea* (negative mode).

Peak (Compound)	Rt (min)	UV-Vis (λ_max_)	LC-MS [M − H]^−^	ESI-IT-MS/MS^n^ Ions	Identification	Reference
**1**	9.58	279	331	313, 271, 211, 193, 169, 125	Monogalloylglucose	[19,20,21,22]
**2**	11.72	271	169	125	Gallic acid	[23,24]
**3**	12.75	274	343	191, 169, 125	3-*O*-galloyl quinic acid	[25]
**4**	17.48	274	325	169, 125	Galloylshikimic acid	[23,26,27,28]
**5**	19.63	280	483	271, 211, 193, 169, 125	Digalloylglucose	[19,20,21,22]
**6**	20.62	279	289	271, 245, 205, 139	(*epi*) catechin	[20,29,30]
**7**	21.52	278	495	343	Digalloylquinic acid	[25]
**8**	23.30	273	801	757, 713, 633, 631, 613	Galloyltannin	[31]
**9**	25.68	278	635	483, 465, 423, 301	Trigalloylglucose	[19,20,21,22]
**10**	26.61	279	787	635, 617, 301	Tetragalloylglucose	[19,20,21,22]
**11**	29.01	277	197	169, 125	Ethyl gallate	[32]
**12**	29.78	279	965	933, 301	Castalagin derivative	[33]
**13**	31.06	257, 365	469	425	Valoneic acid dilactone	[34,35]
**14**	31.94	278	785	633, 301	hexahydroxydiphenyl-digalloylglucose acid	[20,36,37]
**15**	32.60	279	939	787, 769, 635, 617	Pentagalloylglucose	[19,20,21,22]
**16**	34.01	279	865	847, 755, 713, 697, 679, 577, 529, 289	Procyanidin trimer	[30,38]
**17**	36.86	276	477	325, 315, 169	Digalloylshikimic acid	[23,26,27,28]
**18**	40.71	254, 365	301	257, 229, 185	Ellagic acid	[39,40]
**19**	42.27	254, 366	447	315, 300	3-*O*-methylellagic acid 4'-*O*-β-d-arabinopyranoside	[40,41]
**20**	43.24	254, 363	461	315	Methylellagic acid rhamnoside	[42]

The UV spectra and fragmentations in the second order spectra (Table 1) show that the ethanolic extract consists mainly of hydrolyzable tannins, when compared with the literature data for these compounds [19,23,39,43].

Gallic acid and several of its derivatives were identified in the extract when compared to the R_t_, UV and MS spectra. Compound **2** was assigned to gallic acid. The deprotonated molecule ion at *m/z* 169 confirmed the presence of this compound [23,24]. The deprotonated molecule ion at *m/z* 197 was identified with an R_t_ of 29.01 min. Second order spectra of this ion led to the formation of the ion product at *m/z* 169, which is related to the loss of CH_2_ = CH_2_ [M − 28 − H]^−^ and the ion fragment at *m/z* 125 [M − 28 − 44 − H]^−^, as well as the loss of CH_2_ = CH_2_ and CO_2_, thus confirming that the compound is ethyl gallate (**11**) [32].

The deprotonated molecule ion at *m/z* 495 (**7**) was identified as digalloyl quinic acid. The second order spectrum shows the lost group galloyl [M − 152 − H]^−^ providing the ion at *m/z* 343. The fragmentation ion at *m/z* 343 yielded other fragments: *m/z* 191, related to the elimination of a galloyl group [M − H − 152]^−^; and the corresponding fragmentation at *m/z* 169 eliminating the quinic acid with the formation of deprotonated gallic acid [25]. 

For galloyl quinic acid (**3**), the same fragments discussed for the ion at *m/z* 343 were observed. The NMR spectrum showed a chemical shift in the mono- and bi-dimensional spectra, similar to Nishimura *et al*. [44], thus characterizing it as the 3-*O*-galloyl quinic acid. The ^13^C NMR in DMSO-*d*_6_, (ppm), 7.0 T: δ 38.7 (C-2), δ 70.9 (C-3), 71.3 (C-4), 73.2 (C-5), 37.3 (C-6), 166.9 (COO^−^), 108.8 (C-2'), 145.3 (C-3'), 138.3 (C-4'), 145.3 (C-5'), 108.8 (C-6'). The ^1^H NMR in DMSO-*d*_6_, (ppm), 7.0 T: δ 2.09 (m, H-2a), 2.17 (m, H-2b), 5.45 (m, H-3), 4.18 (m, H-4), 3.76 (dd, 2.2 and 2.2 Hz, H-5), 2.00 (m, H-6a), 2.18 (m, H-6b), 7.09 (2H, s, H-2' e H-6'). Correlations observed in the HMBC experiment: H-2a/C-3; H-3/COO^−^; H-5/C-4; H-2'/C-3'; H-2'/C-4'; H-2'/COO^−^; H-6'/C-5'; H-6'/C-4'; H-6'/COO^−^.

The deprotonated molecule ion at *m/z* 325 (**4**) yielded the product ion at *m/z* 169 [M − 156 − H]^−^, which is related to the loss of shikimic acid. Compound **17** showed the deprotonated molecule ion at *m/z* 477. In the second order spectrum, the loss of a galloyl unit [M − 152 − H]^−^ was observed, which yielded the ion at *m/z* 325. Compounds **4** and **17** were identified as mono- and di-galloyl shikimic acid, respectively [23,26,27,28].

Compound **14** displays the deprotonated molecule ion at *m/z* 785. The MS^2^ experiments show the formation of the ion at *m/z* 633 [M − 152 − H]^−^, thus indicating the removal of a galloyl group. The ion at *m/z* 615 was attributed to the elimination of a galloyl unit and a loss of the water molecule [M − 152 − 18 − H]^−^. The ion at *m/z* 483 was attributed to the elimination of the HHDP (hexahydroxydiphenyl) group [M − 301 − H]^−^, and the ion at *m/z* 301 is due to the loss of digalloylglucose [M − 483 − H]^−^ [20,36,37].

The deprotonated molecule ion at *m/z* 801 (**8**) revealed the loss of CO_2_ [M − 44 − H]^−^, which provided the fragmentation at *m/z* 757. MS^3^ spectra of *m/z* 757 yielded the ion at *m/z* 713, which was attributed to the loss of other CO_2_ [M − 88 − H]^−^. These ions led to the suggestion that the molecule is a gallotannin [31]. 

Analysis of Compound **12** shows the deprotonated molecule ion at *m/z* 965, which produced an ion at *m/z* 933 in the MS^2^experiment and in the MS^3^experiment showed a fragment ion at *m/z* 301. Additionally, fragments were observed at *m/z* 915 and 897. The fragments at *m/z* 933, 915 and 897 are typical of the castalagin derivative [33].

**Figure 2:**
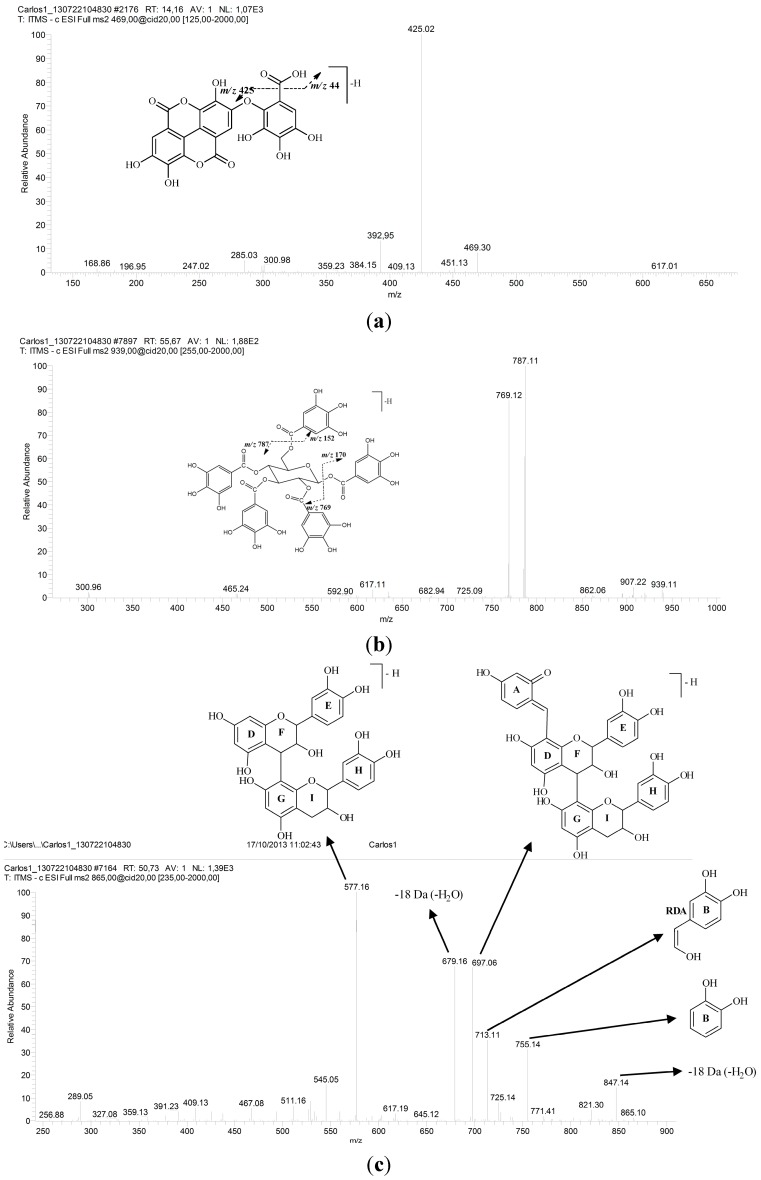
Second-generation product ion spectra obtained for the main precursor ions produced in the FIA-ESI-MS experiment, as well as the proposed fragmentation: (**a**) valoneic acid dilactone; (**b**) pentagalloylglucose; and (**c**) procyanidin trimer. For the conditions, see the Experimental Section.

Analysis of the ions at *m/z* 939, 787, 635, 483 and 331 (**1**, **5**, **9**, **10** and **15**, respectively) showed the presence of a homologous series of galloylglucose. The UV spectra of these compounds shows a λ_max_ = 278–280 nm. MS^2^ experiments with the precursor ions at *m/z* 939 led to product ions at *m/z* 787 attributed to a loss in the galloyl group [M − 152 − H]^−^ and the loss of a water molecule [M − 152 − 18 − H]^−^, which yielded the ion fragment at *m/z* 769. This fragmentation suggests the presence of pentagalloylglucose (Figure 2b). Subsequent analysis of another galloylglucose derivative showed consecutive losses of 152 Da and 170 Da. Therefore, it was possible to clearly identify the tetragalloylglucose (*m/z* 787), trigalloylglucose (*m/z* 635) and digalloylglucose (*m/z* 483) derivatives. In the MS^2^ experiments of the ion *m/z* 331, the main fragments were determined by: the loss of water at *m/z* 313 [M − H − 18]^−^, removal of one and two formaldehyde groups (CH_2_O) from the glucose at *m/z* 271 [M − H − 60]^−^ and *m/z* 211 [M − H − 60 − 60]^−^, respectively, and also by the loss of another water molecule at *m/z* 193 [M − H − 2 × 60 − 18]^−^ and the loss of glucose [M − H − 162]^−^, which leads to the ion at *m/z* 169. The compound was identified as monogalloylglucose [19,20,21,22].

Ellagic acid and its derivatives were also identified in the *C*. *ferrea* extract. The Compounds **13**, **18**, **19** and **20** showed the deprotonated molecule ion at *m/z* 301, 447, 461 and 469, respectively.

The substances displayed UV spectra with λ_max_ of 254 and 365 nm and fragmentation patterns characteristic of ellagic acid derivatives, with losses of 44 Da (CO_2_) and 28 Da (CO) [19]. The ions were detected from the precursor ion *m/z* 301 of the ellagic acid (**18**), which resulted in the ions *m/z* 257 [M − H − CO_2_]^−^, *m/z* 229 [M − H − CO_2_ − CO]^−^, and *m/z* 185 [M − H − 2CO_2_ − CO]^−^ [39,40]. From the identification of ellagic acid, it was possible to check for other derivatives. 

Compound **19** showed the [M − H]^−^ ion at *m/z* 447, which, upon fragmentation, produced ions at *m/z* 315 and 300, through the elimination of a pentose [M − 132 − H]^−^ and a methyl group [M − 132 − 15 − H]^−^ [40,41]. 

The mono-dimensional NMR spectra of this compound (**19**) showed chemical shifts similar to Nono *et al*. [45], which is why it was identified as 3-*O*-methylellagic acid 4'-*O*-β-d-arabinopyranoside. The ^13^C NMR in DMSO-*d*_6_, (ppm), 7.0 T: δ 140.2 (C-3), 110.6 (C-5), 159.8 (C-7), 148.9 (C-4'), 110.6 (C-5'), 159.3 (C-7'), 61.3 (3-OMe) for aglycone; 103.3 (C-1''), 73.4 (C-2''), 76.0 (C-3''), 69.7 (C-4''), 66.3 (C-5'') for the sugar moiety. The ^1^H NMR in DMSO-*d*_6_, (ppm), 7.0 T: δ 7.46 (s, H-5), 7.51 (s, H-5'), 4.04 (3H, s, 3-OMe) for aglycone; 4.98 (d, 7,8 Hz, H-1''), 3.37 (m, H-2''), 3.29 (m, H-3''), 3.42 (m, H-4''), 3.84 (m, H-5''a), 3.35 (m, H-5''b) for the sugar moiety. Correlations observed in the HMBC experiment: H-5/C-3; H-5/C-4; H-5/C-6; H-5/C-7; H-5'/C-3'; H-5'/C-4'; H-5'/C-6'; H-5'/C-7'; H-OCH_3_/C-3.

Compound **20** showed the molecule of the precursor ion at *m/z* 461 [M − H]^−^. This led to ion fragments at *m/z* 315 [M − 146 − H]^−^, which was attributed to the loss of rhamnose and the loss of a methyl group [M − 146 − 15 − H]^−^ at *m/z* 300. The compound was identified as methyl ellagic rhamnoside acid [42]. 

The molecule of the precursor ion at *m/z* 469 (**13**) and second order spectra showed a loss of CO_2_ [M − 44 − H]^−^, which provides the fragment ion at *m/z* 425; see Figure 2a [34,35]. The mono- and bi-dimensional NMR spectra of this compound showed chemical shifts similar to Silva *et al*. and Barakat *et al*. [34,35]. Thus, it was considered to be valoneic acid dilactone. The ^13^C NMR in DMSO-*d*_6_, (ppm), 7.0 T: δ 114.3 (C-1), 105.6 (C-2), 150.0 (C-3), 140.5 (C-4), 109.3 (C-5), 135.6 (C-6), 158.9 (C-7), 112.0 (C-1'), 109.1 (C-2'), 149.0 (C-3'), 140.1 (C-4'), 111.3 (C-5'), 135.9 (C-6'), 159.6 (C-7'), 115.0 (C-l''), 139.1 (C-2''), 139.8 (C-3''), 134.6 (C-4''), 142.9 (C-5''), 109.4 (C-6''), 167.0 (COOH). The ^1^H NMR in DMSO-*d*_6_, (ppm), 7.0 T: δ 6.97 (s, H-5), 7.40 (s, H-5'), δ, 6.99 (s, H-6''). Correlations observed in the HMBC experiment: H-5/C-1; H-5/C-3; H-5/C-5; H-5/C-7; H-5'/C-1'; H-5'/C-3'; H-5'/C-7'; H-6''/C-2''; H-6''/C-4''; H-6''/C-5''; H-6''/COOH. 

The analyses of the UV spectra showed that another class of substances identified in the ethanolic extract from the bark of *C*. *ferrea* is flavan-3-ol (**16** and **6**, Table 1). We observed the trimer (**16**) and monomer (**6**) of this class. ESI-MS analysis showed the presence of the deprotonated molecule ion at *m/z* 865 (Figure 2c). Second-order fragmentation of the precursor ion [M − H]^−^ at *m/z* 865 produced fragments at *m/z* 755 [M − 110 − H]^−^ and can result in the loss of a heterocyclic ring fission (HRF) [29]. The fragmentation of this compound was demonstrated by Hamed *et al*. [30]. Second-order spectra made it possible to verify the formation of the ion at *m/z* 755 [M − 110 − H]^−^ related to the benzene furan fission of the lower part of the trimer, which provided the structure of the dihydroxybenzene (ring B). MS^2^ of the precursor ion at *m/z* 865 also produced a fragment ion at *m/z* 713, generated by retro-Diels-Alder fragmentation (ring C) of the upper part of the trimer, with a loss of 152 Da and consequent elimination of the dimer unit. The product ions of *m/z* 847 and *m/z* 289 were generated from the fragmentation of the precursor molecular ion *m/z* 865, attributed to the loss of water [M − 18 − H]^−^ and to the elimination of the deprotonated catechin (epi) molecule through formation of quinone methide in ring D of the central unit of the trimer [M − 576 − H]^−^. The dimer was not observed in the HPLC-ESI-IT-MS experiment. Therefore, the ion at *m/z* 577 refers to the trimer fragment. In the HPLC-ESI-IT-MS experiment, catechin was observed at Peak **6** with R_t_ = 20.62 and λ_max_ = 279 nm and presented the precursor ion at *m/z* 289, which was attributed to the deprotonated molecule ion [M − H]^−^. MS^2^ of the ion led to the product ion of *m/z* 271, which is related to the loss of a water molecule [M − 18 − H]^−^ and to the characteristic fragments of *m/z* 245 (loss of CO_2_), *m/z* 205 and *m/z* 139 (a break in ring A of the flavan-3-ol compound) [20], which are formed by the retro-Diels–Alder fragmentation mechanism [29].

### 2.2. Validation Method 

**Figure 3:**
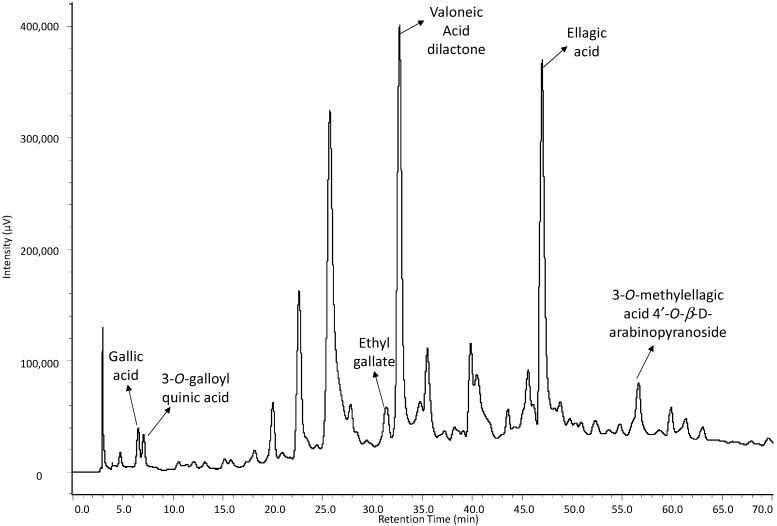
HPLC-DAD chromatogram of the ethanolic extract of the stem bark from *C*. *ferrea*.

Although the separation of compounds could be achieved in the analysis by HPLC/ESI-IT-MS, there was an extensive chromatographic band between R_t_ = 25 min and R_t_ = 38 min, probably due to the high degree of polymerization of compounds in the sample, which resulted in poor chromatographic resolution. Therefore, in the HPLC-DAD analysis, a new chromatographic elution gradient was designed in order to minimize this effect, to improve the peak’s resolution and, consequently, the area calculation for the peaks. With this in mind, one alternative found was to replace Solvent B, which is why methanol was replaced by acetonitrile (Figure 3). The quantification curves were obtained by nine dilutions of the stock solution in a concentration range of 500 to 1.95 µg·mL^−1^ for gallic acid and eight dilutions of the stock solution in a concentration range of 333 to 2.6 µg·mL^−1^. Chromatographic peak areas were calculated for each concentration, and interpolated values were determined as a function of concentration by using linear regression.

Both curves showed good linearity. The following *r*^2^ values were obtained: *r*^2^ of gallic acid = 0.9999 (regression curve: y = 34178x − 105370); and *r*^2^ of ellagic acid = 1.0 (regression curve: y = 169688x − 1257.8). The method presented good linearity over the concentration range evaluated (Table 2). The limit of detection (LOD) for gallic acid was 0.78 μg·mL^−1^, and the limit of quantification (LOQ) was 2.35 μg·mL^−1^. For ellagic acid, the LOD was calculated to be 0.46 μg·mL^−1^, and the LOQ was 2.60 μg·mL^−1^.

**Table 2:** Summary of validation data for ethanolic extract from the stem bark of *C*. *ferrea*.

Property	Ethanolic Extract	
	Gallic Acid	Ellagic Acid
**Linear range (μg·mL^−1^)**	500–1.95	333–2.60	
**Calibration equation**	y = 34178x − 105370	y = 169688x − 1257.8	
**Correlation coefficient (*r*^2^)**	0.9999	1.0	
**LOQ (μg·mL^−1^)**	2.35	2.60	
**LOD (μg·mL^−1^)**	0.78	0.46	
**Interday precision (%RSD)**	4.76	3.66	
**Intraday precision (%RSD)**	0.59	0.76	
**Selectivity**	Selective	Selective	

The overall intraday variations in retention times for the standards were 0.59% for gallic acid and 0.76% for ellagic acid. Interday variations were less than 4.76% for gallic acid and less than 3.66% for ellagic acid. These results indicate that the method had good repeatability, low variability and, thus, good precision. Selectivity was evaluated by comparing the retention time and UV spectra of each standard reference compound with that of the peaks in the *C*. *ferrea* extract (Figure 3).

In order to determine the most practical method, we used two reference standards available on the market. From initial viewing of the chromatogram, it became evident that the main constituents were phenolic acid derivatives. Some derivatives were expressed as gallic acid, while other derivatives were expressed as ellagic. This analysis could be performed because the electronic UV spectra of the standards are practically superimposable with those of the analyzed substances at the monitored wavelength. The concentration of the compound’s chromatographic peak in the extract of the stem bark of *C*. *ferrea* was estimated, and the quantitative analysis results are reported in Table 3.

**Table 3:** Estimation of the contents of phenolic acid derivatives in ethanolic extract from the stem bark of *C*. *ferrea*, expressed by the use of linear regression data of gallic acid (GA) and ellagic acid (EA).

Compound	Concentration ± SD (µg·mL^−1^)	Standard
**Gallic acid**	17.68 ± 0.10	GA
**3-*O*-galloyl quinic acid**	13.26 ± 0.28	GA
**Ethyl gallate**	28.26 ± 0.81	GA
**Valoneic acid dilactone**	63.00 ± 0.93	EA
**Ellagic acid**	57.64 ± 1.22	EA
**Methylellagic acid-pentoside**	4.98 ± 0.06	EA

The results presented in Table 3 are in agreement with the quantification parameters evaluated for the method. For the six quantified substances, the contents are higher than the LOQ, but are within the ranges of its respective standard calibration curve. Therefore, the results show that the method is sensitive enough for analysis of the gallic and ellagic acid derivatives identified in the ethanolic extract from the stem bark of *C*. *ferrea*.

### 2.3. Mutagenic Activity

Carcinogenicity and mutagenicity are among the toxicological endpoints that pose the greatest concern for human health; thus, they are the object of intense research activity and recognized regulatory testing methods [46]. Several reliable and widely used mutagenicity tests were considered for use in this investigation; for example, the micronucleus test and the single cell gel electrophoresis (comet) assay [47,48]. However, the Ames test is used worldwide as an initial screen to determine the mutagenic potential of new chemicals and drugs. The *Salmonella* and Ames tests are *in vitro* models of chemical carcinogenicity and consist of a range of bacterial strains, which together are sensitive to a large array of DNA-damaging agents [46,49]. The four *S*. *typhimurium* strains used in this assay were TA97a, TA98, TA100 and TA102. Strains TA97 and TA98 are used to detect mutagens that cause frameshift mutations, while the TA100 and TA102 strains detect mutagens that cause DNA base-pair substitutions [49,50]. 

Table 4 shows the mutagenic activity detected in the Ames test, both in the presence and absence of microsomal activation. The responses are expressed as revertants/plate, and the mutagenic ratio is in parentheses. The ethanolic extract had no detectable mutagenicity.

**Table 4:** Mutagenic activity expressed by means and standard deviations of the number of revertants/plates and mutagenicity index (MI) (value given in brackets) in TA98, TA100, TA102 and TA97a of *Salmonella typhimurium* treated with different concentrations of ethanol extract from the stem bark of *C*. *ferrea*, with (+S9) and without (−S9) metabolic activation.

Treatments	Number of Revertants (M ± SD)/Plate and MI							
	TA 98		TA 100		TA 102		TA 97a	
mg/plate	−S9	+S9	−S9	+S9	−S9	+S9	−S9	+S9
**0.00 ^a^**	57 ± 3	39 ± 5	128 ± 39	104 ± 3	401 ± 27	369 ± 33	75 ± 5	110 ± 14
**0.26**	68 ± 12(1.2)	40 ± 3(1.0)	130 ± 18(1.0)	121 ± 8(1.2)	434 ± 37(1.1)	414 ± 6(1.1)	109 ± 8(1.5)	145 ± 9(1.3)
**0.52**	70 ± 7(1.2)	38 ± 1(1.0)	135 ± 2(1.1)	116 ± 1(1.1)	425 ± 42(1.1)	414 ± 15(1.1)	84 ± 18(1.1)	166 ± 5(1.5)
**1.04**	65 ± 7(1.1)	38 ± 6(1.0)	159 ± 8(1.2)	121 ± 13(1.2)	394 ± 16(1.0)	409 ± 17(1.1)	93 ± 10(1.2)	151 ± 7(1.4)
**1.56**	66 ± 14(1.2)	38 ± 4(1.0)	142 ± 13(1.1)	118 ± 10(1.1)	393 ± 20(1.0)	407 ± 17(1.1)	91 ± 9(1.2)	165 ± 10(1.5)
**2.08**	61 ± 5(1.1)	37 ± 3(1.0)	152 ± 11(1.2)	102 ± 2(1.0)	392 ± 21(1.0)	403 ± 8(1.0)	81 ± 9(1.1)	164 ± 29(1.5)
**C+**	797 ± 79 ^b^	2204 ± 255 ^e^	1193 ± 39 ^c^	1229 ± 94 ^e^	1192 ± 49 ^d^	1804 ± 43 ^e^	716 ± 74 ^b^	2636 ± 132 ^e^

M ± SD = mean ± standard derivation of number of revertants/plate. Negative control: ^a^ dimethylsulfoxide (100 μL/plate). Positive controls (C+): ^b^ 4-nitro-o-phenylenediamine (10 µg/plate); ^c^ sodium azide (2.5 µg/plate); ^d^ mitomycin (3 µg/plate), without S9; and ^e^ 2-antramine (0.125 µg/plate) with S9.

Given that medicinal plants have been contributing to health globally, it is imperative to evaluate their safety in terms of the mutagenic potential of their constituent compounds over both the short and long term. Short-term tests that detect genetic damage have provided information on the carcinogenic risk to humans for various chemicals [49].

In Brazil, a large number of herbal extracts are used in folk medicine to treat various types of disease. *C*. *ferrea* is a medicinal plant that is extensively used. In the present study, mutagenic activity assays with *Salmonella* demonstrated that the ethanolic extract from the stem bark of *C*. *ferrea* is not mutagenic. The absence of a mutagenic effect is important to the traditional system of medicine, supports a growing interest in the pharmacological evaluation of this plant and is a positive step forward in determining the safe use of this plant.

## 3. Experimental

### 3.1. Chemicals and Reagents

Trifluoroacetic acid (TFA), acetonitrile and methanol were of HPLC grade and purchased from the Tedia Company (Fairfield, OH, USA). All other reagents used were of analytical grade. The following standards were used for quantitative analysis: gallic acid, ellagic acid and ethyl gallate, which were purchased from Sigma-Aldrich (São Paulo, Brazil). Water was purified with a Milli-Q system (Millipore, Billerica, MA, USA). All solutions prepared for HPLC were filtered through a 0.22-µm GHP (hydrophilic polypropylene) filter (Waters, Milford, MA, USA) before use.

### 3.2. Plant Material

Stem bark from *C*. *ferrea* was collected in September, 2011, in Juína (11°22'40"S and 58°44'27"W) in Mato Grosso, Brazil, and authenticated by Márcia Cleia Vilela dos Santos from the Federal University of Mato Grosso (UFMT), Sinop, MT, Brazil. A voucher specimen (No. 3021) was deposited at the herbarium of the Herbário Centro Norte Mato-grossense (CNMT).

### 3.3. Extraction

Dried and powdered stem bark (1610.0 g) of *C*. *ferrea* was macerated, at room temperature, with ethanol (4×, 48 h). The solution was evaporated under vacuum to give 260.09 g (16.2%) of crude ethanolic extract from the stem bark.

### 3.4. Isolation and Identification of the Compounds

A portion of ethanolic extract (500 mg) was redissolved in MeOH-H_2_O (2:8 *v*/*v*) and subjected to medium pressure liquid chromatography (MPLC), a pumping system equipped with a Buchi^®^ C-601, using a C18 column (16 cm × 3.0 cm × 50 µm) and with a 5.0 mL/min flow. The mobile phase consisted of water (Eluent A) and methanol (Eluent B). The isocratic mode afforded 7 fractions (Fr). Compounds **3**, **13** and **19** were obtained from the Fractions Fr1 (10% A), Fr3 (30% A) and Fr6 (50% A), respectively. The compounds were purified by a semi-preparative HPLC-DAD Jasco PU 2086 series pumping system equipped with a Jasco MD-2010 PDA detector and Chromnav software. The Fractions (Fr1 = 135 mg; Fr2 = 44 mg; and Fr3 = 28 mg) were separated by semi-preparative HPLC-DAD, using a Phenomenex Synergi Hydro RP 18 column (250 mm × 10 mm i.d.; 10 µm) and a Rheodyne 100 µL manual injector loop, at a flow rate of 6.0 mL·min^−1^. The mobile phase consisted of water (Eluent A) and methanol (Eluent B) containing 0.1% acetic acid. The following elution gradient was applied: 0–30 min for 0%–20% B (Fr1); 0–30 min for 25%–35% B (Fr2); and 0–30 min for 40%–50% B (Fr3). This afforded the following compounds: 3-*O*-galloyl quinic acid (**3**, 13.4 mg); valoneic acid dilactone (**13**, 3.6 mg); and 3-*O*-methylellagic acid 4'-*O*-β-d-arabinopyranoside (**19**, 9.6 mg). 

The isolated compounds were analyzed and identified by NMR, UV and MS experiments and then compared with data from the literature. The ^1^H-NMR and ^13^C-NMR 1D and 1H-NMR 2D-NMR ^13^C *g*-HMBC experiments were performed on a Bruker^®^ 300 MHz (7.0 T) nuclear magnetic resonance spectrometer. For sample preparation for the NMR experiments, dimethyl sulfoxide (DMSO-*d*_6_, Cambridge Isotope Laboratories, Inc., Andover, MA, USA) was used.

For the compounds (**2**, **11**, **18**), we opted to develop a methodology based on analysis by HPLC**-**DAD, in an attempt to enable direct identification of secondary metabolites using co-injection of authentic standards (Sigma-purity of 98.5%). For the chromatographic condition, see Section 2.7.

For the other compounds identified in the ethanolic extract from the stem bark of *C*. *ferrea*, the characterization and identification of the peaks are shown in the Table 1, where UV-Vis absorptions and electrospray ionization mass spectrometry in negative ion mode of all the compounds detected are reported and compared with the literature data.

### 3.5. HPLC/ESI-IT-MS Analyses

The ethanol extracts of *C*. *ferrea* (stem bark) were analyzed by on-line HPLC/ESI-IT-MS, using a SURVEYOR MS micro system coupled in-line to an LCQ Fleet ion-trap mass spectrometer (Thermo Scientific). HPLC separation was conducted on a Phenomenex (Torrance, CA, USA) Synergi Hydro RP18 (250 × 4.6 mm i.d.; 4 μm), at a flow rate of 0.8 mL·min^−1^. The mobile phases utilized were H_2_O (A) and MeOH (B). In both of these phases, acetic acid (0.1%) was added. Gradient elution was applied for 75 min. The initial condition was 5% (B), which was increased until reaching 100% (B) at 70 min and then held at 100% (B) for an additional 5 min. 

The column effluent was split into two by means of an in-line T junction, which sent it to both ESI-MS and UV-DAD; 80% was sent to the UV-DAD detector and 20% was analyzed by ESI-MS in negative ion mode with a Fleet LCQ Plus ion-trap instrument from Thermo Scientific. The capillary voltage was set at −20 kV, the spray voltage at −5 kV and the tube lens offset at 100 V. The sheath gas (nitrogen) flow rate was set to 80 (arbitrary units), and the auxiliary gas flow rate was set at 5 (arbitrary units). Data were acquired in MS scanning modes. The capillary temperature was 275 °C. Xcalibur 2.1 software (Thermo Scientific, Waltham, MA, USA) was used for data analysis.

### 3.6. FIA-ESI-IT-MS^n^ Analyses

The ethanolic extract was dissolved in methanol and injected by Flow Injection Analysis (FIA) into the ESI source (flow rate of 10 µL·min^−1^). Each ion, corresponding to each peak of the LC-MS chromatogram, was analyzed in the negative ESI-MS mode. Nitrogen was used both as a drying gas, at a flow rate of 60 (arbitrary units), and as a nebulizing gas. The ion spray voltage was 5 kV, and the tube lens offset was −55 V. The nebulizer temperature was set to 275 °C, and a potential of −4 V was used on the capillary. Negative ion mass spectra were recorded in the *m/z* range of 150–2000. The first event was a full-scan mass spectrum to acquire data on ions in the *m/z* range. The second event was an MS/MS experiment in which data-dependent scanning was performed on deprotonated molecules of the compounds. The collision energy for MS/MS was adjusted to 20%–30% and an activation time of 30 ms.

### 3.7. Quantification by HPLC-DAD of the Ethanolic Extract Obtained from the Stem Bark 

The ethanolic extract obtained from the stem bark of *C*. *ferrea*, as well as the standards, were analyzed on a high performance liquid chromatograph (HPLC). The system used was a JASCO 2010 (Jasco, Tokyo, Japan) equipped with a PU-2089S Plus pump, a MD-2018 Plus Photodiode Array Detector (PDA), an AS-2055 Plus auto sampler and CO-2065 plus column oven. The analytical column was a Phenomenex (Torrance, CA, USA) Synergi Hydro RP18 (250 × 4.6 mm i.d.; 4 μm) equipped with a Phenomenex security guard column (4.0 × 2.0 mm i.d.). The flow-rate was 1.0 mL·min^−1^, and 20 μL was injected in each analysis. All chromatographic analyses were performed at 22 °C and monitored at λ = 214, 279 and 365 nm. The mobile phase was composed of water (Eluent A) and acetonitrile (Eluent B), both containing 0.1% TFA. The following elution gradient was applied: 0–15 min for 5%–10% B; 15–65 min for 10%–25% B; and 65–70 min for 25%–100% B. ChromNav software (Workstation JASCO ChromNav 1.18.03) was used to control the analytical system and to collect and process data.

### 3.8. Preparation of Samples and Standards for Analysis by HPLC-DAD

The extract (20 mg) was weighed in a 2.0-mL flask, dissolved in 0.5 mL of DMSO (dimethylsulfoxide), brought to volume with methanol and then placed in an ultrasonic bath for 10 min. 

The ellagic acid standard (1.0 mg) was dissolved in 3 mL of methanol:DMSO (50:50 *v*/*v*). This stock solution was used to prepare the remaining ellagic acid solutions included in the calibration curve. The samples of standard gallic acid (0.5 g) were dissolved, separately, in 1 mL of methanol. Methanol was used for subsequent dilutions.

The standard solutions and the crude extract were filtered through a 0.22-µm PTFE (Millex^®^) filter before analysis by HPLC-DAD.

### 3.9. Identification of Peaks 

To characterize the chemical constituents (**2**, **11** and **18**) or isolated compounds from the ethanolic extract of *C*. *ferrea* stem bark (**3**, **13** and **19**), the retention times of each peak in the HPLC-DAD chromatograms were analyzed, along with UV spectra data and mass fragmentation patterns, and they were compared with literature data. Compounds purified by chromatography revealed a purity of 95%–99%.

### 3.10. Quantitative Determination of Constituents

The quantification of substances **2**, **3**, **11**, **13**, **18** and **19** was done using the external standard method. The quantification of individual constituents was performed using a regression curve, each point in triplicate. Measurements were performed at 254 nm, which is the maximum absorbance.

### 3.11. Linearity, Detection Limit, Quantification Limit and Precision

The calibration curves were obtained based on eight (ellagic acid) and nine (gallic acid) levels of concentration of standard mixtures. Chromatogram peak areas at 254 nm were plotted against the known concentrations of the standard solutions, in order to establish the calibration equations. The stock solutions were dissolved separately in spectroscopy grade methanol in order to obtain solutions that were appropriately diluted for each of the substances to furnish the following concentrations: 1.95, 3.91, 7.81, 15.62, 31.25, 62.5, 125, 250 and 500 µg·mL^−1^ for gallic acid and 2.6, 5.2, 10.4, 20.81, 41.64, 83.25, 166.5 and 333 µg·mL^−1^ for ellagic acid. Each solution was analyzed in triplicate.

A linear least squares regression of the peak areas as a function of the concentrations was performed to determine the correlation coefficients. The equation parameters (slope and intercept) of each standard curve were used to obtain the concentration values for the samples. The LOD and LOQ were calculated based on the standard deviation of the y-intercept (σ) and the slope of the calibration curve (S), which were obtained from linear regression. The LOD was calculated using the expression 3.3 σ/S, and the LOQ was calculated using 10 σ/S. The precision was expressed as a relative standard deviation (RSD). The areas under curves and retention times of the three consecutive injections, performed at each concentration and on three different days, were used to calculate interday precision (% RSD). Intraday precision data for peak areas and retention times were calculated from six non-consecutive injections, performed at each concentration on the same day.

### 3.12. Salmonella/Microsome Assay

Mutagenicity assays were performed by pre-incubating test extracts for 20–30 min with the *Salmonella typhimurium* strains TA100, TA98, TA97a and TA102, either with or without metabolic activation [50]. *S*. *typhimurium* strains were kindly provided by Dr. B. Ames, University of California, Berkeley, CA, USA. The S9-mix was freshly prepared before each test, with an Aroclor-1254-induced rat liver fraction purchased (lyophilized) from Moltox (Molecular Toxicology Inc.). The metabolic activation system consisted of 4% of the S9 fraction, 1% of 0.4 M MgCl_2_, 1% of 1.65 M KCl, 0.5% of 1 M D-glucose-6-phosphate disodium, 4% of 0.1 M NADP, 50% of 0.2 M phosphate buffer and 39.5% sterile distilled water. Five different doses of the test extract (0.26, 0.52, 1.04, 1.56 and 2.08 mg/plate) were diluted with DMSO, the concentrations of which were selected based on a preliminary toxicity test. In all subsequent assays, the upper limit of the dose range was either the highest non-toxic dose or the lowest toxic dose determined in the preliminary assay. The toxicity was apparent either as a reduction in the number of His^+^ revertants or as an alteration in the auxotrophic background (*i.e*., the background lawn). The various concentrations of tested compounds were added to 500 µL of buffer (pH 7.4) and 100 µL of bacterial culture and incubated at 37 °C for 20–30 min. Then, 2 mL of top agar were added to the mixture, which was subsequently poured onto a plate containing minimum agar. The plates were incubated at 37 °C for 48 h, and the His+ revertant colonies were counted manually. The influence of metabolic activation was tested by adding 500 µL of the S9 mixture in place of the buffer. All experiments were performed in triplicate.

The standard mutagens used as positive controls in experiments without the S9 mix were 4-nitro-o-phenylenediamine (10 µg/plate) for TA98 and TA97a, sodium azide (2.5 µg/plate) for TA100 and mitomycin C (3 µg/plate) for TA102. 2-Anthramine (0.125 µg/plate) was used in experiments with metabolic activation of all strains. DMSO served as the negative (solvent) control. The results were analyzed with the Salanal statistical software package (U.S. Environmental Protection Agency, Monitoring Systems Laboratory, Las Vegas, NV, version 1.0, from Research Triangle Institute, RTP, NC, USA) [51], adopting the model of Bernstein *et al*. [52]. The data (revertants/plate) were assessed by variance analysis (ANOVA), followed by linear regression. The mutagenic index (MI) was also calculated for each dose as the average number of revertants per plate divided by the average number of revertants per plate of the negative (solvent) control. A sample was considered to be positive when MI ≥ 2 for at least one of the tested doses and if the response was dose dependent [16,53].

## 4. Conclusions

In this study, a comprehensive identification of chemical composition was conducted using the HPLC/ESI-IT-MS and FIA-ESI-IT-MS^n^ methods, with a total of twenty compounds being identified. Additionally, a quantitative validation method was developed for the simultaneous determination of the six compounds, which were derivatives of the ellagic acid and gallic acid. The absence of a mutagenic effect in the ethanolic extract from the stem bark of *C*. *ferrea* is important for traditional medicine, supports a growing interest in the pharmacological evaluation of this plant and is a positive step forward in determining the safe use of this species.
